# Long locking plate combined with locking attachment plate in patients with periprosthetic femoral fracture around ipsilateral stem after total knee arthroplasty

**DOI:** 10.1186/s12891-023-06726-x

**Published:** 2023-07-20

**Authors:** Oog-Jin Shon, Seung Jae Cho, Gi Beom Kim

**Affiliations:** 1 Department of Orthopedic Surgery, Yeungnam University College of Medicine, 170 Hyeonchung-Ro Nam-Gu, Daegu, 42415 Republic of Korea; 2 Department of Orthopedic Surgery, Yeungnam University Medical Center, 170 Hyeonchung-Ro Nam-Gu, Daegu, 42415 Republic of Korea

**Keywords:** Total knee arthroplasty, Periprosthetic femoral fracture, Stemmed Implantation, Long Locking Plate, Locking Attachment Plate

## Abstract

**Background:**

The purpose of this study was to introduce the surgical technique using long locking plate and locking attachment plate (LAP) in patient with periprosthetic femoral fracture around ipsilateral stem after total knee arthroplasty (TKA). Moreover, we sought to investigate the outcomes of this fixation technique and to propose a new subtype in the existing classification of periprosthetic femoral fractures.

**Methods:**

From January 2013 to January 2022, thirty-four consecutive periprosthetic femoral fractures around ipsilateral stem following TKA with minimum 1-year follow-up were enrolled in this study. Most cases were fixed with long-locking plate and LAP using the MIPO technique. For subgroup analysis, we classified patients with stemmed hip implant (group H) and stemmed knee implant (group K). Bone union, American Knee Society Score (AKSS) scale, Knee Injury and Osteoarthritis Outcome Score for Joint Replacement, the Western Ontario and McMaster Universities Osteoarthritis Index for pain and function, and range of motion were investigated.

**Results:**

The number of group H and K were 24 patients (70.6%) and 10 patients (29.4%), respectively. The mean age at operation was 71.5 years (range, 65‒85 years), and the mean follow-up period was 27.5 months (range, 12‒72 months). Bone union was confirmed radiographically in all patients, and the mean union time was 4.9 months (range, 3.5‒6 months). There were no significant differences in radiographic and clinical outcomes between the groups.

**Conclusions:**

Long-locking plate combined with LAP showed favorable radiographic and clinical outcomes in patients with periprosthetic femoral fracture around ipsilateral stem after TKA.

**Level of evidence:**

Level IV, Retrospective Case Series.

## Introduction

Recently, as the number of primary total knee arthroplasty (TKA) increases, the number of revision TKA using stemmed implants is increasing. In particular, approximately 5,000 revision TKAs are implemented annually in Korea alone [1]. Treatment of periprosthetic femoral fracture following TKA is more challenging due to poor bone stock, existing implants, and bone cement. Meanwhile, periprosthetic femoral fracture after total hip arthroplasty (THR) or proximal femoral intramedullary (IM) nail, which is basically equipped with a stem, is complex and requires high-level surgical skills [2]. Since the presence of stemmed implants may impede stability and securing of sufficient bone stock for fixation [3], the fixation strategy in the case of stemmed implantation may be insufficient with only conventional lateral plating [4, 5].

Although supplementary fixation methods including cerclage wiring or cable have been introduced, these fixatives lack enough resistance to torsional stress, and relative high failure rates have been reported [6, 7]. Accordingly, the Locking Attachment Plate (LAP® 3.5 mm for LCP 4.5 mm; DepuySynthes; Synthes GmbH, Oberdorf, Switzerland) that can be used in combination with the standard long locking plates to build a stable construct around the stem was developed [5, 8].

To the best of our knowledge, some authors have proposed several classifications of periprosthetic femoral fracture following TKA [9–12]. However, there has been paucity of literatures regarding the fractures around the stemmed implant and their treatment. Moreover, few classifications suggested fixation strategies according to fracture subtype.

The purpose of the present study was to introduce the surgical technique using long locking plate and LAP in patients with periprosthetic femoral fracture around ipsilateral stem after TKA. Moreover, we sought to investigate the outcomes of this fixation technique and to propose a new subtype in the existing classification of periprosthetic femoral fractures. We hypothesized that this fixation strategy would have a favorable outcome in patients with periprosthetic femoral fractures around the stem after TKA.

## Patients and methods

### Patients’ demographic characteristics

**Fig. 1:**
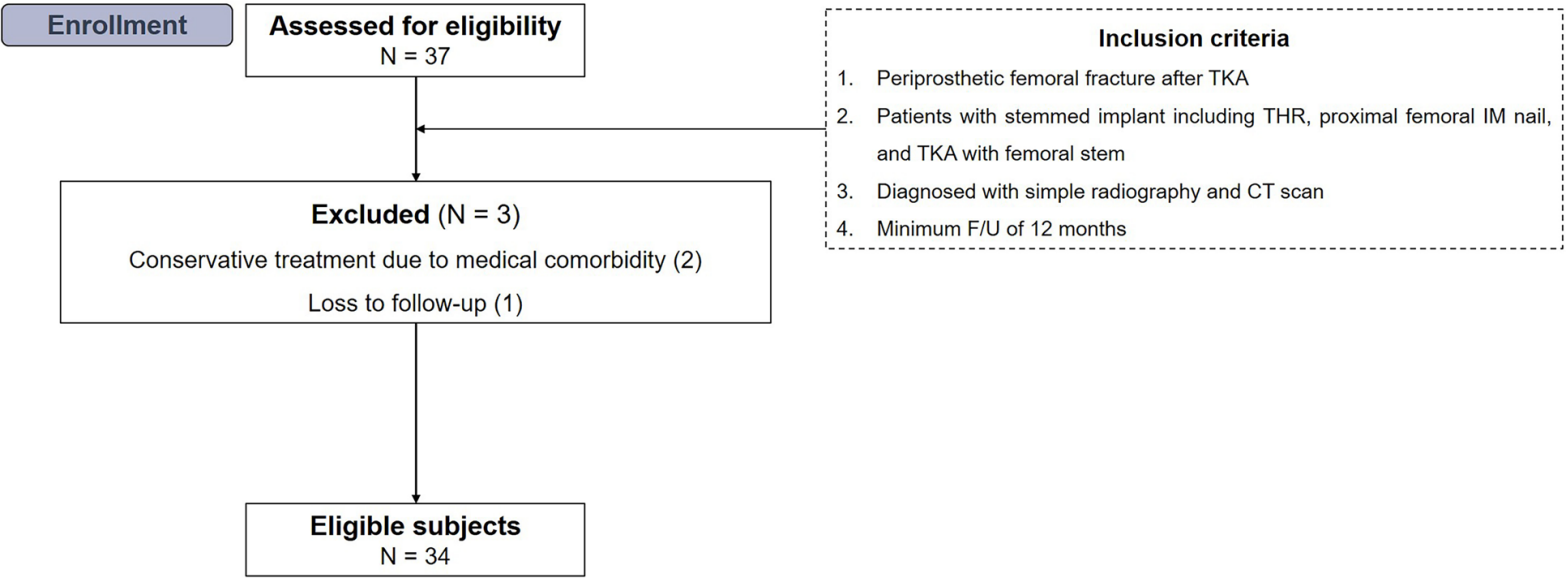
Flow diagram illustrating patient enrollment. Overall, 34 knees were enrolled in the present study

From January 2013 to January 2022, 37 consecutive patients who underwent periprosthetic femoral fractures around ipsilateral stem after TKA were assessed for eligibility. The fracture type and final diagnosis were made through preoperative simple radiograph and computed tomography (CT) scan by two independent board-certified orthopedic surgeons. The inclusion criteria of the current study were as follows: (1) periprosthetic femoral fracture in patients with stemmed implant after TKA including THR, proximal femoral IM nail, and TKA with femoral stem, (2) patients who underwent operative treatment using long-lateral locking plate with LAP, and (3) a minimum follow-up of 12 months. From a total of 37 patients, 3 were excluded for the following reasons: (1) conservative treatment due to medical comorbidity (*n* = 1) and (2) inadequate follow-up period (< 12 months, *n* = 2). Finally, 34 patients were enrolled in the current study (Fig. 1). The study was approved by the institutional review board our hospital.

### Surgical techniques (stemmed hip implant)

**Fig. 2:**
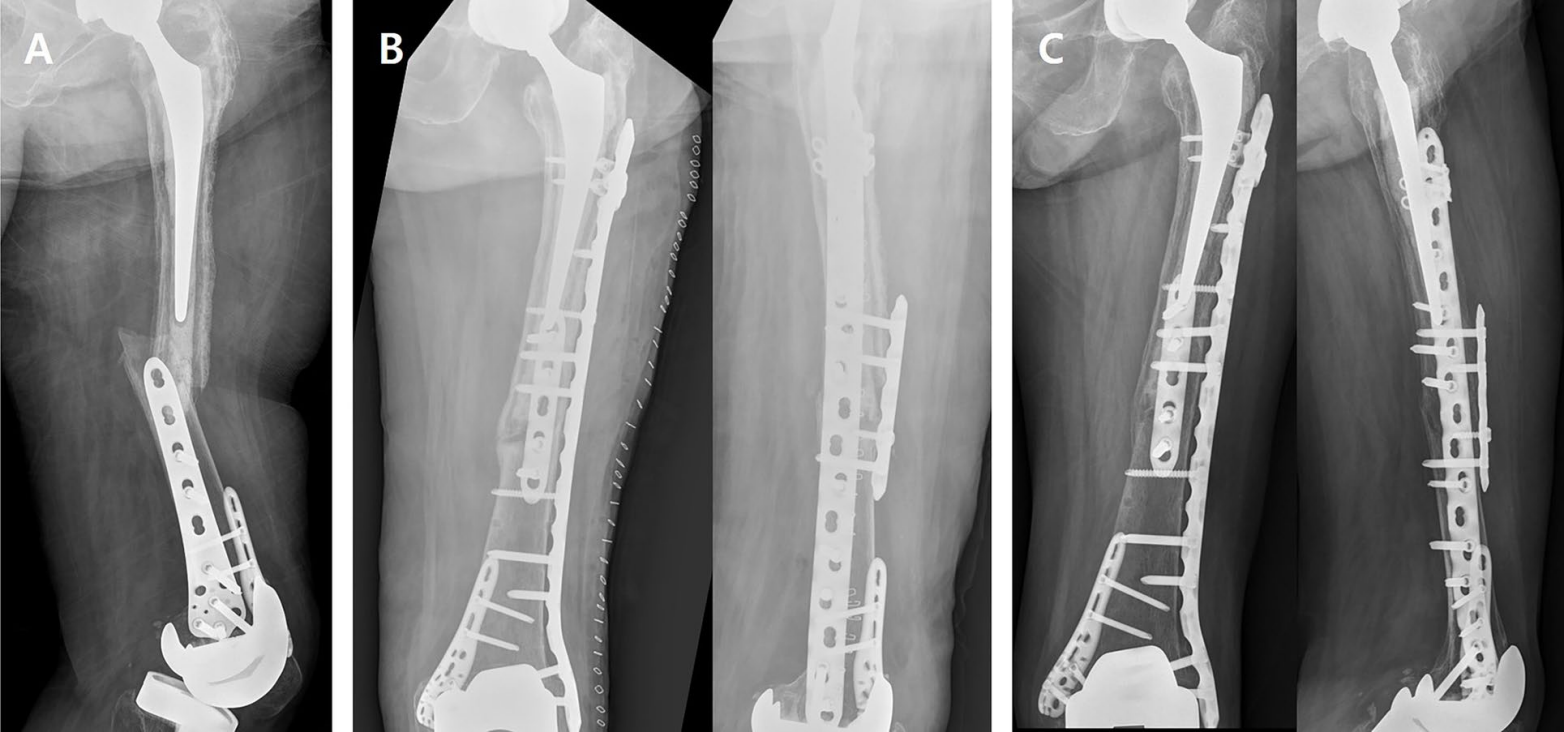
**A** A preoperative plain radiograph of a 68-year-old female who sustained a periprosthetic femoral shaft fracture following THA, representing a Vancouver type C. A 4.5 mm short locking plate fixation was initially used to reduce the fracture. The patient had already undergone dual-locking plate fixation for an extremely distal femoral periprosthetic fracture after TKA, 3 years ago. **B** Pre-existing anatomical short locking plate (DF plate) was removed. The patient was treated with a 4.5-mm LCP long broad-curved plate overlapping the zone of the stem at least 6 cm. Intraoperative plate bending was performed using a large bender to fit the proximal and distal femoral geometry. Supplementary fixation using a LAP was performed in the overlapped zone. **C** At 5 months postoperatively, a solid bone union was acquired. THA, total hip arthroplasty; TKA total knee arthroplasty; DF, distal femur; LCP, locking compression plate; LAP, locking attachment plate

All operations were performed using the same technique by two experienced-orthopedic surgeons in a single center. Periprosthetic femoral fractures following TKA in patients with stemmed hip implants were classified as group H. Most periprosthetic femoral fractures around stem are fixed using a minimally invasive plate osteosynthesis (MIPO) technique with a lateral locking plate (LCP Distal Femur Plate or 4.5-mm LCP long broad-curved plate; DepuySynthes GmbH, Oberdorf, Switzerland). The length of the plate was set to overlap the zone of the stem at least 6 cm by referring to previously reported biomechanical studies [13]. Since an anatomically pre-contoured locking plate (LCP Distal Femur Plate) had limited length options available, a 4.5-mm LCP long broad-curved plate was used when overlap with the stem zone was insufficient. When using the long broad-curved plate, intraoperative contouring was performed using a large bender to fit the proximal and distal femoral geometry by a surgeon. Additionally, since it was difficult to secure enough bone stock around the hip implant, the overlapped zone of the stem with plate was supplemented with LAP (Fig. 2).

### Surgical techniques (stemmed knee implant)

**Fig. 3:**
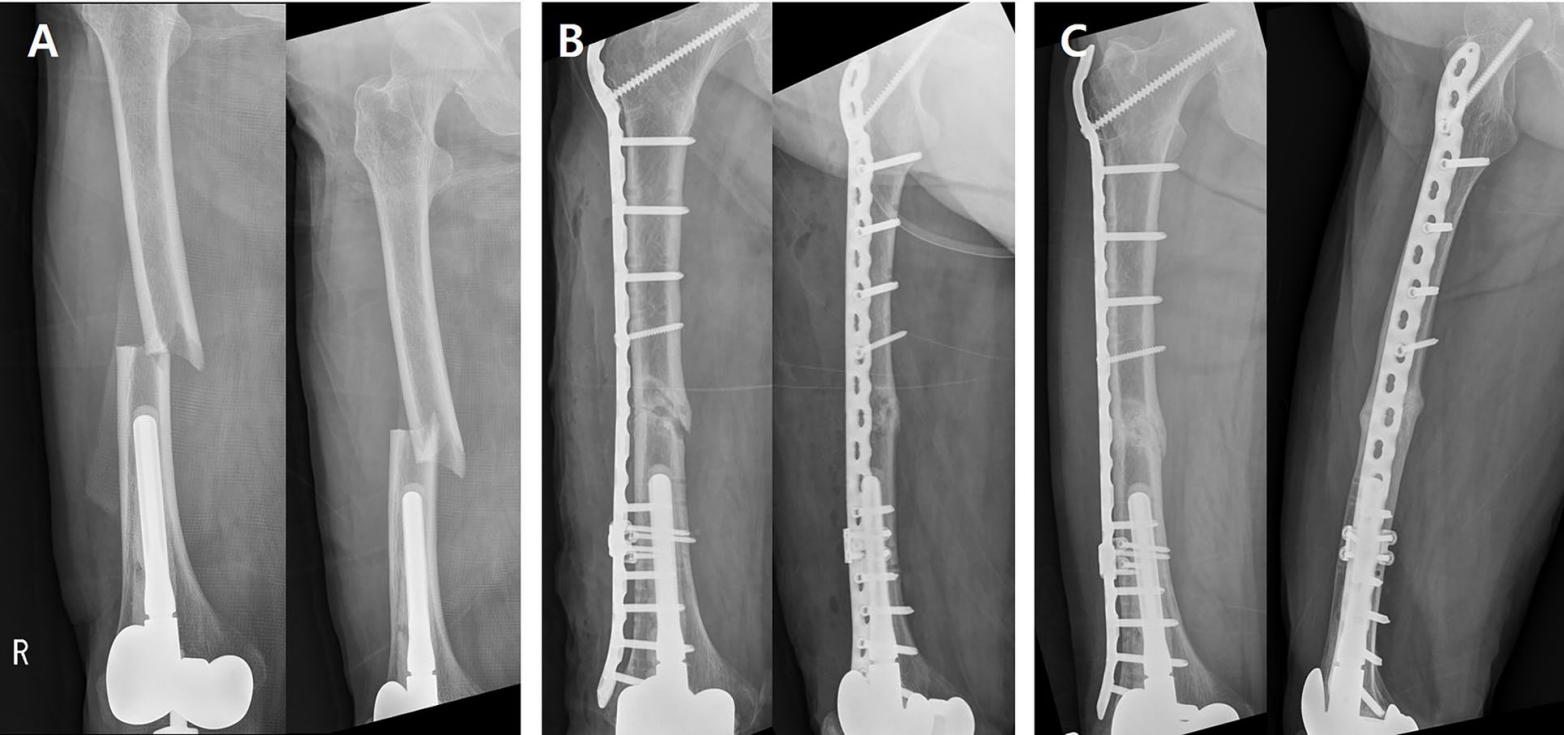
**A** Preoperative anteroposterior and lateral plain radiographs of an 83-year-old female who sustained a periprosthetic femoral shaft atypical fracture following TKA with long stem, representing a Su type I. **B** The patient was treated with a 4.5-mm LCP long broad-curved plate overlapping the zone of the stem at least 6 cm. Intraoperative plate bending was performed using a large bender to fit the proximal and distal femoral geometry. Supplementary fixation using a LAP was performed in the overlapped zone. Fixation of the femoral neck was additionally performed using a 4.5-mm long cancellous screw to protect the entire femur from future fracture. **C** At 4 months postoperatively, a solid bone union was acquired. TKA, total knee arthroplasty; LCP, locking compression plate; LAP, locking attachment plate

**Fig. 4:**
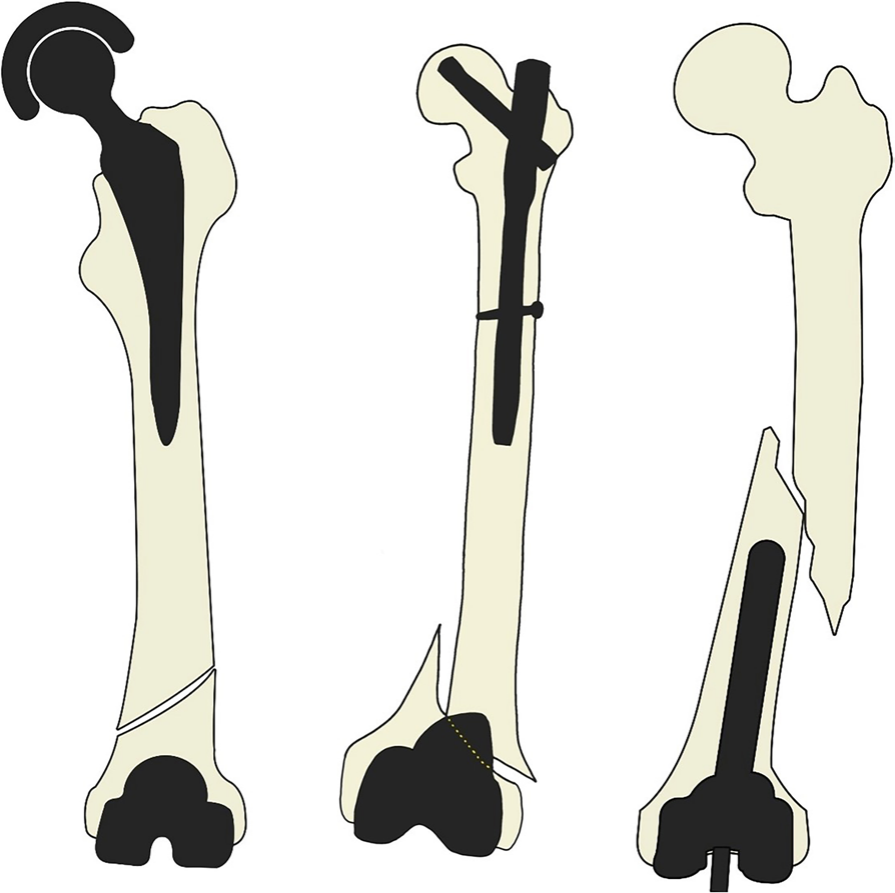
Schematic representation of new subtypes of periprosthetic femoral fracture after TKA, in cases with stemmed implants. **A** THA (equivalent of Vancouver type C), **B** proximal femoral IM nail, and **C** TKA with stem-mounted femoral component. THA, total hip arthroplasty; IM, intra-medullary; TKA, total knee arthroplasty

Periprosthetic femoral fractures around a stemmed knee implant after TKA were classified as group K, and fixed with a long-locking plate (LCP Distal Femur Plate or 4.5-mm LCP broad-curved plate) through the MIPO technique. Considering the fracture level, to avoid the most proximal tip of the lateral plate acting as a stress riser around the lesser trochanter, the plate length was set such that a 4.5-mm LCP broad-curved plate covered the greater trochanter. Intraoperative contouring was performed using a large bender to fit the proximal and distal femoral geometry by a surgeon. In this case, fixation of the femoral neck was additionally performed using a 4.5-mm long cancellous screw to protect the entire femur from future fracture (Fig. 4). An anatomically pre-contoured locking plate was difficult to apply because the length options available were limited and the stiffness was too strong to bend. Otherwise, the pre-contoured locking plate was used. Since it was difficult to secure sufficient bone stock for screw fixation in the overlapped zone with stem, LAP was used to supplement. According to the length of the stem, 1 or 2 LAPs were attached on the existing lateral plate and additional small locking screws were inserted (Fig. 3). In only one case where the fracture line extended to medial side, the medial buttress was performed using an anatomical locking plate for the proximal humerus (PHILOS; DepuySynthes GmbH, Oberdorf, Switzerland) through a subvastus approach.

### Rehabilitation protocol

The same rehabilitation protocol was applied to all patients regardless of the operator. No cast or immobilizer was applied. Active and passive postoperative range of motion (ROM) exercise was allowed on the second or third day of surgery after removal of the suction drain. Partial weight-bearing with a crutch was allowed at six weeks postoperatively. Full weight-bearing was allowed at 3 or 4 months after surgery after radiographic bone union was confirmed.

### Outcome assessments

Demographic characteristics were investigated before surgery. Anteroposterior (AP) and lateral radiographs of the whole femur including the hip and knee joints were performed preoperatively; at 6 weeks and 3, 6, and 12 months postoperatively; and then every year until the last follow-up. All computed tomography (CT) scans were taken preoperatively, and obtained with SOMATOM Definition AS + (Siemens, Germany) using a bone algorithm. Radiographic measurements were retrieved using a picture-archiving and communication system (Maroview®, version 5.4; Marotech, Seoul, Korea) in format of DICOM (Digital Imaging and Communicating in Medicine), and radiographic measurements were performed at regular follow-ups by an independent researcher. The fracture pattern was based on the preoperative simple radiographs and CT scans. Except for periprosthetic fractures around the hip IM nail, fractures around hip arthroplasty were classified as Vancouver classification [14], and fractures around stemmed knee arthroplasty were classified as Su classification [10].

The primary outcome was bone union. Bone union was radiographically defined as the formation of a bridging callus across the fracture line on 4 cortices based on serial radiographs, and clinically defined as pain-free during weight-bearing on the operated side [15].

Clinical evaluations were documented using the American Knee Society Score (AKSS) scale (knee score and function score) [16], the Knee Injury and Osteoarthritis Outcome Score for Joint Replacement (KOOS-JR) [17], and the Western Ontario and McMaster Universities Osteoarthritis Index (WOMAC) for pain and function [18]. They were assessed by an independent investigator in outpatient clinic. ROM of the knee joint (including flexion contracture and further flexion angle) was measured using a long-armed goniometer by an independent researcher. Clinical assessments were performed in all patients at latest follow-up.

Moreover, complication rates including union-related problems (such as delayed union or non-union), refracture, loosening of stemmed implants, and infection were investigated.

### Statistical analysis

Statistical evaluation was performed using IBM SPSS software (Version 28.0; IBM Corporation, Armonk, NY, USA). All dependent variables were tested for normality of distribution and equality of variances using the Kolmogorov–Smirnov test and analyzed using parametric or nonparametric tests based on normality. Continuous data were expressed as means with SDs, and analyzed using the Mann–Whitney U-test to assess the differences in clinical and radiographic variables between the groups. Categorical variables were analyzed using the Fisher’s exact test. For all tests, the statistical significance was set at *p* < 0.05. Reliabilities for all radiographic classification were analyzed using intraclass correlation coefficients, and reliabilities were classified as little if any (correlation coefficient, ≤ 0.25), low (0.26 – 0.49), moderate (0.50 – 0.69), high (0.70 – 0.89), or very high (≥ 0.90).

## Results

In total, 34 patients (26 women and 8 men) were included in this study. The mean age at operation was 71.5 years (range, 65‒85 years), and the mean follow-up period was 27.5 months (range, 12 ‒ 72 months) (Table 1). Incidences of group H and K were 70.6% (24/34) and 29.4% (10/34), respectively. Among group H, there were 20 patients with Vancouver type B1 (5, 14.7%) and C (15, 44.1%). Remaining 4 patients (11.8%) had proximal femoral IM nail. There were 10 patients with Su type I (7, 20.6%) and type II (3, 8.8%) (Table 2).

**Table 1:** Patients’ Characteristics

Variable	No. (%) or Mean (range)
Patients, n	34
Sex, n (%)^a^	
Female	26 (76.5)
Male	8 (23.5)
Age, yr^b^	71.5 (65 ‒ 85)
BMI, kg/m^2 b^	23.9 (17.7 ‒ 33.3)
Osteoporosis, n (%)^a^	20 (58.8)
Mean follow-up, mo^b^	27.5 (12.0 ‒ 72.0)
Diagnosis of TKA^a^	
OA	31 (91.2)
RA	2 (5.9)
Post-traumatic OA	1 (2.9)
Injury mechanism^a^	
Slip down	28 (82.4)
Fall	3 (8.8)
Traffic accident	3 (8.8)
Mean time to injury after TKA, mo^b^	47.4 (9.0 – 108.0)

*BMI* Body mass index, *TKA* Total knee arthroplasty, *OA *Osteoarthritis, *RA* Rheumatic arthritis, *THA* Total hip arthroplasty

^a^Data are presented as numbers (percentages)

^b^Data are presented as mean (range)

**Table 2.**
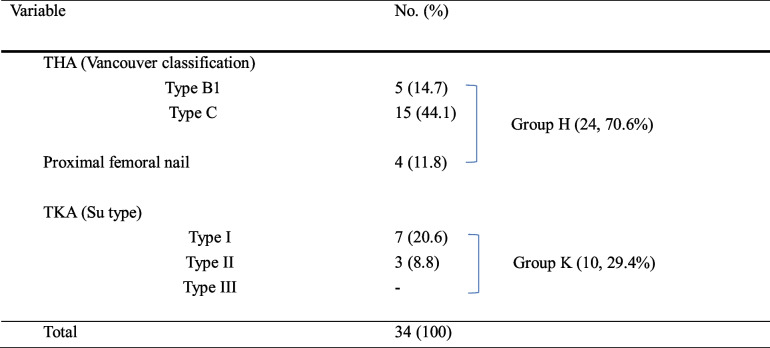
Previous implantation and relevant classification

*THA* Total hip arthroplasty, *TKA* Total knee arthroplasty.

Data are presented as numbers (percentages).

We obtained fracture healing without loss of reduction in all patients. Bone union was confirmed radiographically and the mean union time was 4.9 months (range, 3.5 ‒ 6.0 months). There were no union-related complications. In clinical outcomes, there were no significant differences between groups. There were no cases of revision hip or knee arthroplasty performed for loosening of stemmed implants at latest follow-up (Table 3). The intra- and inter-observer reliabilities for radiographic evaluations are shown in Table 4. All measurements showed very high intra- and inter observer reliabilities.

**Table 3:** Radiographic and clinical outcomes

Variable	No. (%) or Mean (range)			
	Total	Group H	Group K	*P* value
		(*n* = 24)	(*n* = 10)	
Mean time to union, mo^a^	4.9 (3.5 ‒ 6.0)	4.8 (3.5 ‒ 6.0)	5.1 (3.8 ‒ 6.0)	0.325
AKSS (latest F/U)^b^				
Knee score	83.4 ± 4.8	83.8 ± 3.8	83.2 ± 5.5	0.861
Function score	81.3 ± 3.4	81.2 ± 2.4	81.7 ± 4.4	0.65
KOOS-JR scores (latest F/U)^b^	43.2 ± 4.1	44.2 ± 4.3	42.1 ± 4.2	0.583
WOMAC (latest F/U)^b^				
Pain	3.2 ± 1.5	3.0 ± 1.6	3.3 ± 1.4	0.734
Stiffness	4.2 ± 1.3	4.0 ± 1.1	4.6 ± 1.4	0.59
Function	8.6 ± 2.3	8.5 ± 2.0	8.9 ± 2.4	0.476
Total	16.6 ± 3.9	16.6 ± 3.9	16.6 ± 3.9	0.612
ROM of the knee joint^b^				
FC (˚)	4.8 ± 6.1	4.2 ± 7.1	5.0 ± 5.6	0.693
FF (˚)	112.2 ± 5.6	114.7 ± 6.1	110.2 ± 7.1	0.49
Complications				-
Delayed or non-union	-	-	-	
Malunion	-	-	-	
Refracture	-	-	-	
Infection	-	-	-	
Revision arthroplasty	-	-	-	

*AKSS* American Knee Society Score, *F/U* Follow-up, *KOO-JR* The Knee Injury and Osteoarthritis Outcome Score for Joint Replacement, *WOMAC* The Western Ontario and McMaster Universities Osteoarthritis Index, *ROM* Range of motion, *FC* Flexion contracture, *FF* Further flexion

^a^Data are presented as mean (range)

^b^Data are presented as mean (standard deviation)

**Table 4:** Intra- and inter-class correlation coefficients of the radiographic evaluations

	Intra-observer	Inter-observer
Vancouver type B1	0.98	0.97
Vancouver type C	0.99	0.99
Su type I	0.99	0.99
Su type II	0.99	0.98
Union period	0.93	0.90

Values presented as absolute values. The data show almost perfect intra- and inter- observer agreement in the radiographic evaluations.

## Discussion

The most notable finding of the present study was that a novel fixation technique using a long-locking plate and LAP showed favorable radiographic and clinical outcomes in periprosthetic femoral fracture in patients with stemmed hip or knee implants after TKA.

The procedural number of revision TKA has grown steadily over the past decade [1]. In this revision scenarios, the stem has been frequently used to aid transfer loads from the compromised articular and metaphyseal bone to the tibial cortical bone and to widely distribute the increased stress of a constrained articulation [19]. Generally, the revision stem enhances mechanical stability through stress shielding, which can be improved by resistance to shear, reduced lift-off, and decreased micromotion [20]. Meanwhile, treatment using proximal femoral nails has been regarded as the gold standard for unstable proximal femoral fractures, which mainly occur in the elderly [21]. As life expectancy increases, the frequency of periprosthetic fractures that occur in situations such as additional hip arthroplasty, proximal femoral IM nail fixation, and stem-mounted femoral component in patients who have already undergone TKA may increase.

For periprosthetic femoral fractures with stemmed implants in the hip or knee joint, IM nailing can be difficult to perform. Accordingly, it is inevitable to apply the locking plate system, but the plate length can be an issue. According to a biomechanical study, an overlap of at least 6 cm was recommended to avoid stress risers caused by insufficient length in periprosthetic or inter-prosthetic femoral fractures [13]. Since it is difficult to secure sufficient bone stock, it may be difficult to obtain sufficient stability with screw fixation belonging to the plate hole. In a biomechanical comparative study of LAP and cerclage wiring, since the LAP enabled positioning of the bi-cortical locking screw laterally of the prosthesis stem, excellent stability for fixation around the stem in periprosthetic fracture was shown [8]. In the present study, the authors performed additional fixation in the overlapped zone with one or two LAPs depending on the length, and no union-related complications occurred in all patients.

Some authors have proposed several classifications of periprosthetic fracture following TKA, based on fracture displacement, level, and quality of bone stock [10–12]. Up to date, although a proposed new classification of locking plate fixation methods according to fracture modalities has been proposed, there has been paucity of literature on periprosthetic fracture around ipsilateral stem after TKA. Moreover, these existing classifications do not reflect the current fixative system such as long-locking plate and LAP. Since the presence of stemmed implants make it difficult to secure sufficient bone stock for screw fixation and can cause concerns with the stability of the fixation [3], the fixation strategy in the case of stemmed implantation may be insufficient with only conventional lateral plating. A recent study proposed a new practical classification of fixation strategies according to fracture patterns in periprosthetic femoral fractures after TKA [9]. The authors reported the need for dual locking plate fixation in extreme distal fractures with medial beak. However, even in this classification, cases with stemmed implants were not considered.

With the exception of patients requiring revision arthroplasty due to loosening of pre-existing components [11], we would propose a new subtype for the classification of periprosthetic femoral fracture after TKA based on the results of the present study: (1) patients who have undergone hip arthroplasty surgeries including THR and hemi-arthroplasty, (2) proximal femoral IM nail, and (3) TKA with stem-mounted femoral component (Fig. 4). Since periprosthetic femoral fractures around stemmed implants need to be treated differently from fractures after primary TKA, it is necessary to divide this type of fracture into another subtype of periprosthetic femoral fracture following TKA.

Despite the informative results of this study, it has some limitations that need to be considered. First, the small cohort and the lack of powerful statistical data may be a major concern. These can be attributed to the fact that the incidence of periprosthetic fracture in patients with stemmed implant was very low. Therefore, a multicenter study with a large sample is needed. Second, since there was no direct comparison with a group treated with different fixatives, it was difficult to guarantee that this fixation method would show better results than other methods. Third, a female predominance was observed in this study. Thus, the same results may be different in populations with different sex ratios. Since the incidence of knee OA and osteoporosis was much higher in women than men in Asia, it has been impossible to avoid this trend [22]. Fourth, since this study used 34 patients and univariate analysis to test the equality of radiographic and clinical outcomes, it needs to be noted that the results could be different in a multivariate analysis conducted in a study such as a randomized study. Lastly, this study had a relatively short follow-up period. Thus, mid- to long-term outcomes were not guaranteed. However, since the primary outcome of this study was to evaluate radiographic bone union, the mean follow-up of 27.5 months (at least 12 months) may be reasonable for orthopedic surgeons to discern the usefulness of this novel fixation technique for periprosthetic femoral fracture after TKA in patients with stemmed implants.

## Conclusion

Long-locking plate combined with LAP showed favorable radiographic and clinical outcomes in patients with periprosthetic femoral fracture around ipsilateral stem after TKA. Since periprosthetic femoral fractures around the stem require such a different technique than conventional fixation, it may be reasonable to classify this type as a new subtype of periprosthetic femoral fracture after TKA.

## Data Availability

The datasets used during the current study available from the corresponding author on reasonable request.
